# pKa Modulation of the Acid/Base Catalyst within GH32 and GH68: A Role in Substrate/Inhibitor Specificity?

**DOI:** 10.1371/journal.pone.0037453

**Published:** 2012-05-25

**Authors:** Shuguang Yuan, Katrien Le Roy, Tom Venken, Willem Lammens, Wim Van den Ende, Marc De Maeyer

**Affiliations:** 1 Laboratory of Molecular Plant Physiology, Institute of Botany and Microbiology, KU Leuven, Heverlee, Belgium; 2 Laboratory for Biomolecular Modelling, Department of Chemistry, Division of Biochemistry, Molecular and Structural Biology, KU Leuven, Heverlee, Belgium; Institute of Enzymology of the Hungarian Academy of Science, Hungary

## Abstract

Glycoside hydrolases of families 32 (GH32) and 68 (GH68) belong to clan GH-J, containing hydrolytic enzymes (sucrose/fructans as donor substrates) and fructosyltransferases (sucrose/fructans as donor and acceptor substrates). In GH32 members, some of the sugar substrates can also function as inhibitors, this regulatory aspect further adding to the complexity in enzyme functionalities within this family. Although 3D structural information becomes increasingly available within this clan and huge progress has been made on structure-function relationships, it is not clear why some sugars bind as inhibitors without being catalyzed. Conserved aspartate and glutamate residues are well known to act as nucleophile and acid/bases within this clan. Based on the available 3D structures of enzymes and enzyme-ligand complexes as well as docking simulations, we calculated the pKa of the acid-base before and after substrate binding. The obtained results strongly suggest that most GH-J members show an acid-base catalyst that is not sufficiently protonated before ligand entrance, while the acid-base can be fully protonated when a substrate, but not an inhibitor, enters the catalytic pocket. This provides a new mechanistic insight aiming at understanding the complex substrate and inhibitor specificities observed within the GH-J clan. Moreover, besides the effect of substrate entrance on its own, we strongly suggest that a highly conserved arginine residue (in the RDP motif) rather than the previously proposed Tyr motif (not conserved) provides the proton to increase the pKa of the acid-base catalyst.

## Introduction

Carbohydrates play an important role in a diverse array of biological processes. Their functional and structural variety implies a large amount of enzymes involved in their modification, synthesis and breakdown. The classification of ‘carbohydrate-active enzymes’ (CAZy) (http://www.cazy.org/) [1], [2] is based on their biological properties, including glycoside polysaccharide lyases, hydrolases, carbohydrate esterases, carbohydrate-binding modules and glycosyltransferases. Glycoside hydrolases (GH) split the glycosidic bond between two carbohydrates or between a carbohydrate and an aglycon moiety. They play important roles both in plants and micro-organisms. In plants for instance, they are involved in carbohydrate partitioning affecting overall growth and development, pollen development and fertilization [3], [4]. GHs are further classified in families. Because there is a direct relationship between sequence and folding similarities [5], such a classification helps to reveal the evolutionary relationships between such enzymes. Families GH32 and GH68 belong to clan GH-J, one of the 14 clans defined in CAZy. Both families contain of 5-fold β-propeller in their tertiary structure, harboring the catalytic site. GH32 members contain an additional C-terminal β-sheet domain absent in GH68. Naumoff proposed that clan GH-J should be combined with α-arabinases and β-xylosidases [5] (families GH43 and GH62) into one “β-fructosidase superfamily”.

**Figure 1:**
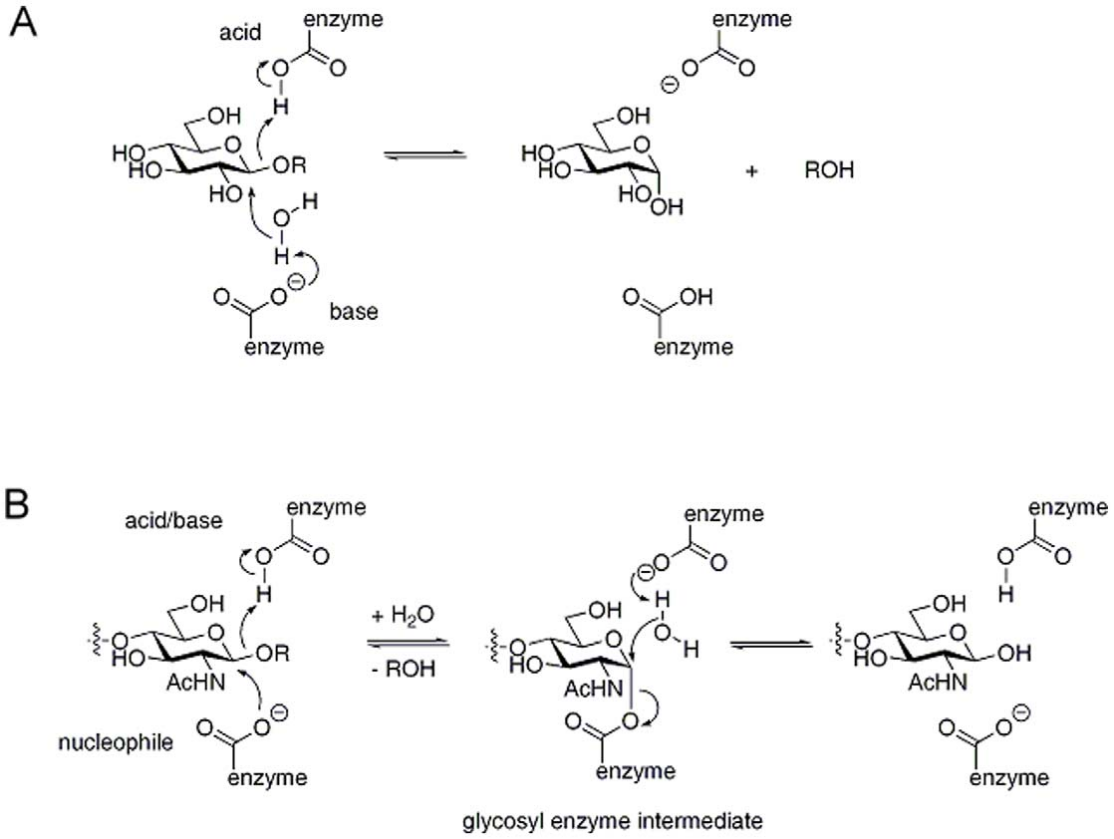
Inverting and retaining catalytic mechanisms in GHs. (A) Inverting mechanism: the conformation of the glycoside residue inverts after reaction (B) Retaining mechanism as occurring in GH32 and GH68: the glycoside residue keeps the same conformation after reaction.

Within GHs, inverting (Figure 1A) and retaining (Figure 1B) mechanisms can be discriminated, depending on the outcome of the reaction [6]. In all cases, the reaction involves two acidic residues. In the inverting mechanism, the configuration of the anomeric carbon is inverted in a single step, using a nucleophile that activates a water molecule (Figure 1A). In contrast, the GHs that retain the configuration of the anomeric carbon operate via a double displacement mechanism in which a covalent glycosyl-enzyme intermediate is formed and hydrolyzed via an oxocarbenium ion-like transition state, which is believed to be stabilized by a transition state stabilizer (Figure 1B). GH32 and GH68 are retaining enzymes using Asp (e.g. WMN**D**PNG motif in GH32) as nucleophile and Glu (e.g. **E**C motif in GH32) as proton donor (acid/base catalyst) as first established for yeast invertase [7]–[10]. As first established for yeast invertase, the process involves the protonation of the glycosidic oxygen by an acid/base catalyst and the attack on the anomeric carbon of the substrate by the nucleophile [10]. The transition state is believed to be stabilized by a transition state stabilizer (an Asp in the R**D**P motif) [11].

GH-J members show extended structural similarities, especially in the vicinity of the active site. However, an enormous variation in substrate specificities is observed in clan GH-J including levansucrases, inulosucrases (GH68), plant and microbial invertases, microbial endo- and exo-type inulinases and levanases and an array of plant fructosyltransferases (1-SSTs, 1-FFTs, 6-SFTs, 6^G^-FFTs) and plant fructan exohydrolases (1-FEHs, 6-FEHs, 6&1-FEHs) [12]. Essentially, these enzymes use sucrose or fructans as donor substrates and sucrose, fructans or water as acceptor substrates. An array of 3-D structures became available within clan GH-J [13]–[25] (see further Table 1 and 2), boosting structure-function research in this area. Substrate specificity is often influenced by one or a few amino-acid substitutions [26]. However, when it comes to substrate specificity, many questions remain, including a full understanding why some sugars act as inhibitors rather as substrates for some enzymes. This suggests that “binding without catalysis” frequently occurs. So far, only one hypothesis has been formulated to explain the absence of catalysis despite binding (e.g. sucrose as inhibitor in 1-FEH IIa, [26]). Deeper insights would greatly contribute to a further rational enzyme design within clan GH-J.

**Table 1:** pKa value of crystallized GH32 family members.

AtcwINV1 of *Arabidopsis thaliana* pH 5 [23]							
	Description	E203	R148	Y279	Ligand	Sub/Inhi^1^	[A^−^]/[HA]
**2AC1**	APO	4.7	14.0	18.6	na^2^	na	2.0
**2QQU**	D239A	3.6	11.7	16.3	Sucrose	Sub	25.1
**2QQV**	E203A	na	11.4	15.1	Sucrose	Sub	na
**2QQW**	D23A	7.5	13.2	17.5	Sucrose	Sub	3×10^−3^
**TRANS**	TS	5.7	14.1	18.6	Fructose	na	0.2
**Dock**	Dock to 2AC1	6.0	13.3	18.6	Sucrose^3^	Sub	0.1

1-FEH IIa of *Cichorium intybus* pH 5 [15]							
	Description	E201	R146	Y274	Ligand	Sub/Inhi	[A^−^]/[HA]
**1ST8**	APO	3.0	13.6	15.2	na	na	100
**2ADD**	Inhibitor	3.3	12.9	15.1	Sucrose	Inhi	50.1
**Dock**	Dock to 1ST8	5.4	12.9	14.1	1-Kestose^3^	Sub	0.3
**Dock**	Dock to 1ST8	5.3	13.0	14.6	Inulobiose^3^	Sub	0.2

Exo-inulinase of *Aspergillus awamori* pH 5 [17], [19]							
	Description	E241	R188	Y313	Ligand	Sub/Inhi	[A^−^]/[HA]
**1Y4W**	APO	3.7	12.8	16.9	na	na	20.0
**Dock**	Dock to 1Y4W	4.7	12.1	16.9	Sucrose^3^	Sub	0.5
**Dock**	Dock to 1Y4W	8.0	11.8	17.3	1-Kestose^3^	Sub	1×10^−3^

β-fructosidase of *Thermotoga maritima* pH 6 [14], [18]							
	Description	E190	R137	Y240	Ligand	Sub/Inhi	[A^−^]/[HA]
**1UYP**	APO	5.0	17.5	16.9	na	na	10.0
**Dock**	Dock to 1UYP	6.5	16.7	16.4	Sucrose^3^	Sub	0.3

Fructosyltransferase of *Aspergillus japonicus* pH 6 [22]							
	Description	E292	R190	Y369	Ligand	Sub/Inhi	[A^−^]/[HA]
**3LF7**	APO	7.9	15.7	18.1	na	na	0.01
**Dock**	Dock to 3LF7	11.1	14.7	18.2	Sucrose^3^	Sub	7.9×10^−6^
**Dock**	Dock to 3LF7	10.8	14.6	18.0	1-Kestose^3^	Sub	1.6×10^−5^
**Dock**	Dock to 3LF7	11.4	14.6	18.2	Nystose^3^	Sub	4.0×10^−6^

Fructofuranosidase of *Schwanniomyces occidentalis* pH 5 [21]							
	Description	E230	R178	Y293	Ligand	Sub/Inhi	[A^−^]/[HA]
**3KF3**	APO	3.8	12.9	17.4	na	na	15.9
**Dock**	Dock to 3KF3	6.6	12.4	17.4	Sucrose^3^	Sub	0.03

β-fructofuranosidase of *Bifidobacterium longum* pH 6 [24]							
	Description	E235	R180	Y302	Ligand	Sub/Inhi	[A^−^]/[HA]
**3PIG**	APO	4.1	13.7	15.1	na	na	79.4
**Dock**	Dock to 3PIG	7.1	13.0	15.4	Sucrose^3^	Sub	0.08
**Dock**	Dock to 3PIG	7.6	12.8	15.5	1-Kestose^3^	Sub	0.03

1 Sub/Inhi: substrate or inhibitor.

2 na: not applicable for this case.

3 ligand docked into related APO crystal structure.

**Table 2:** pKa value of crystallized GH68 family members.

Levansucrase of *Bacillus subtilis* pH 6 [13], [20]							
	Description	E342	R262	Y411	Ligand	Sub/Inhi^1^	[A−]/[HA]
**1OYG**	APO	5.9	9.6	14.4	na^2^	na	1.26
**Dock**	Dock to 1OYG	8.5	9.8	13.6	Sucrose^3^	Sub	0.003
**Dock**	Dock to 1OYG	8.0	12.0	14.3	Raffinose^3^	Sub	0.01

Levansucrase of *Bacillus megaterium* pH 7 [25]							
	Description	E352	R256	Y421	Ligand	Sub/Inhi	[A−]/[HA]
**3OM6**	APO	4.8	15.9	15.0	na	na	158.5
**Dock**	Dock to 3OM6	7.0	15.5	15.9	Sucrose^3^	Sub	1.0

Levansucrase of *Gluconacetobacter diazotrophicus* pH 5 [16]							
	Description	E401	R308	Y484	Ligand	Sub/Inhi	[A−]/[HA]
**1W18**	APO	1.7	14.3	20.9	na	na	1995.3
**Dock**	Dock to 1W18	4.9	13.2	20.8	Sucrose^3^	Sub	1.3

1 Sub/Inhi: substrate or inhibitor.

2 na: not applicable for this case.

3 ligand docked into related APO crystal structure.

Proton donors (acids) and acceptors (bases) provide and accept protons during catalytic processes. This involves pKa changes of residues around the catalytic pocket [7], [9]. Here, it is described that the protonation state of the acid/base catalyst, as resolved by pKa calculation, is not favorable for catalysis in many GH-J members. However, this can be changed upon substrate entrance. A Tyr next to the acid/base was proposed to modulate the pKa values of the acid/base [27], but pKa calculations and Molecular Dynamics simulations in this work indicate that an Arg next to the acid/base is a more suitable candidate. This Arg (**R**DP motif) is completely conserved in clan GH-J while the Tyr (**Y**ASK motif) is not [28]. Similar findings have been reported before in another enzyme clan [29]. Based on all these findings, an improved catalytic mechanism is proposed within the GH-J clan.

## Results

### AtcwINV-1: existing enzyme-sucrose complexes and sucrose docking

**Figure 2:**
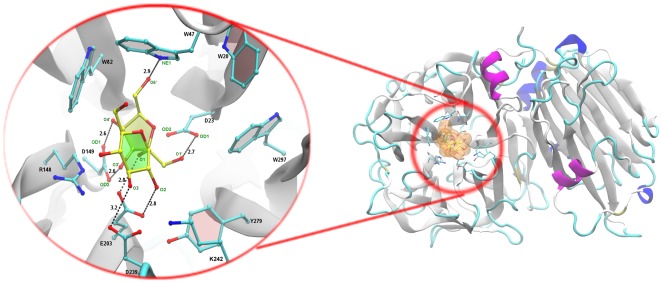
Structure of AtcwINV1 docked with sucrose. Right panel: global view of the structural organization of AtcwINV1. Left panel: a detailed view on the active site showing the position of docked sucrose (yellow) and its interactions with neighbouring amino-acids. Distance units are in angstrom (Å).

**Figure 3:**
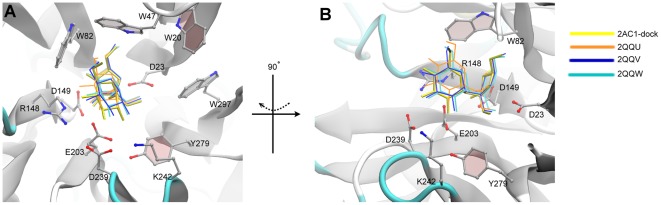
Superimposed AtcwINV1-sucrose complexes with docked sucrose. AtcwINV1-sucrose complexes were derived from Lammens et al. (2008) [30] and compared to the position of docked sucrose (Figure 2). Two views (A,B) are presented. B is obtained after 90° rotation around the vertical axis. Yellow: native AtcwINV-1 docked with sucrose; Orange: crystal structure of mutated AtcwINV1 (D239A, PDB: 2QQU) with sucrose; Blue: crystal structure of mutated AtcwINV1 (E203A, PDB: 2QQV) with sucrose; Cyan: crystal structure of mutated AtcwINV1 (D23A, PDB: 2QQW) with sucrose. While the position of the fructose moiety is conserved, the position of the glucose moiety showed more variation.

**Figure 4:**
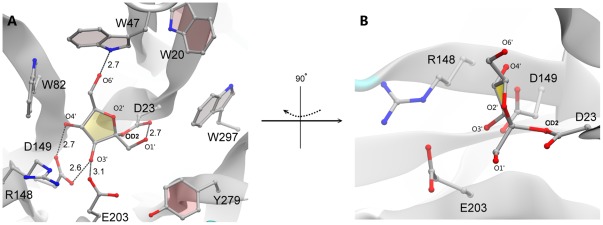
Estimation of the transition state of AtcwINV1. (A) Simulation of the covalent enzyme-fructosyl transition state in AtcwINV1. A covalent bond is formed between fructose and the nucleophile D23. Distance units are in angstrom (Å). (B) This view is generated by 90° rotation around the vertical axis.

The GH32 member AtcwINV-1, the most important cell wall invertase (sucrose as preferential donor) in the model plant *Arabidopsis thaliana*, became one of the best structurally studied GH-J enzymes, since various mutated and inactive AtcwINV-1/sucrose complexes with similar sucrose binding positions were obtained next to the apo-enzyme (2AC1) [30]. We checked the predictive power of sucrose docking simulations in this system and indeed confirmed that Glide [31], [32] in Schrödinger 2011 could position sucrose in a correct way in the active site of AtcwINV1, as observed in the mutated crystal structures 2QQU, 2QQV and 2QQW (Figures 2 & 3). Moreover, a simulation of the covalent enzyme-fructosyl intermediate (Figure 4) was obtained by using Schrödinger 2011.

### The substrate binding mode in AtcwINV1

In the docked AtcwINV1 (2AC1)/sucrose complex, the sucrose molecule is stabilized by a series of H-bonds including a 2.8 Å H-bond between the glycosidic oxygen O1 and acid/base E203, a distance allowing proton transfer from E203 to the glycosidic oxygen. A 2.7 Å H-bond is observed between the O1′ of the fructose part of nucleophile D23. Two 2.6 Å H-bonds between O3′/O4′ and the transition state stabilizer D149 are observed. Further, two additional H-bonds are found between the O6′ of fructose and the nitrogen in the W47 side chain (2.9 Å) and between the O2 of Glc and E203 (2.8 Å) (Figure 2). Moreover, D239 forms a weak H-bond with O3 of Glc (3.2 Å). Superimposing the docking result with the mutated AtcwINV-1/sucrose complexes [30] shows that the fructose moiety of sucrose takes an identical position while some slight variation is observed among the complexes for the position of glucose (Figure 3). Molecular dynamics (MD) studies confirmed the stability of the conserved H-bond distances of this binding mode (Figure S1).

### Simulating the enzyme-fructosyl transition state

**Figure 5:**
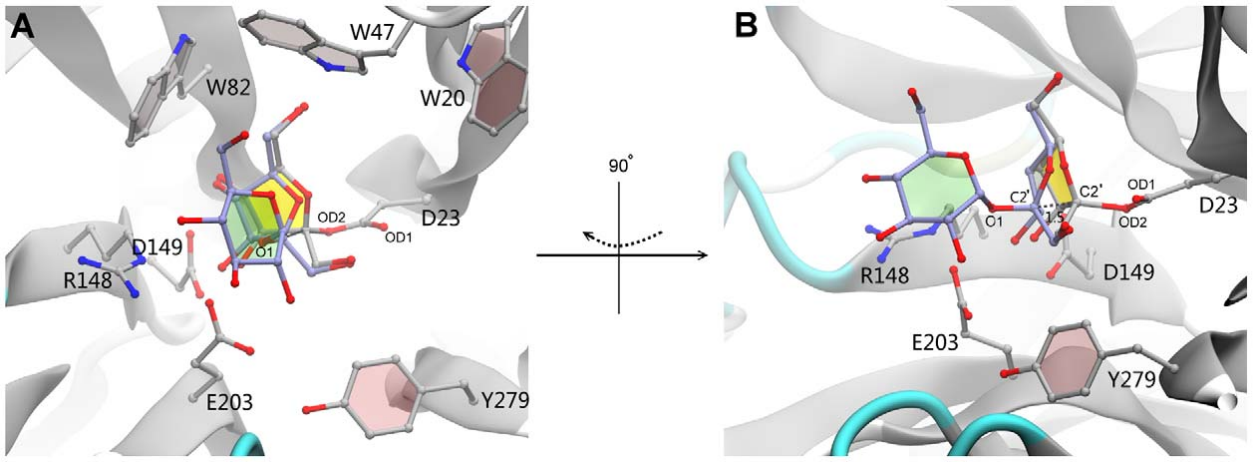
Superimposed AtcwINV1/sucrose and transition state. (A) Superimposal of AtcwINV1/docked sucrose (as derived from Figure 2) and the covalent enzyme-fructosyl transition state (derived from Figure 4). Gray: transition state; ice-blue: sucrose. (B) is generated by 90° rotation around the vertical axis. Distance units are in angstrom (Å).

The covalent transition state is stabilized by several strong H-bonds (Figure 4): 2.6 Å and 2.7 Å H-bonds are found between O3′ and O4′ in the covalently bound fructose and the transition stabilizer D149; O6′ and O1′ show H-bonds with W47 (2.7 Å) and D23 (2.7 Å) respectively; a weak H-bond is found between O3′ and the acid/base E203. The position of the covalent fructose in the transition state is rather similar to the one in the active AtcwINV1/sucrose complex (Figure 5A). However, the position of C2 in the transition state shifted 1.5 Å to the inner part of the active pocket (Figure 5B). Compared to the active AtcwINV1/sucrose complex, the conformation of C2′-OD2 flipped 180° which is in agreement with the double displacement mechanism [6].

### Relation between pH and pKa. pKa calculation of amino acids in proteins

An acid dissociation constant, Ka, is a quantitative measure of the strength of an acid in solution. It is the equilibrium constant for the acid-base/dissociation reaction. Three methods are available to calculate the pKa of amino-acid residues in proteins: methods based on the Poisson-Boltzmann equation (PBE), empirical methods and MD-based methods [33]–[35]. We applied the most widely used pKa calculation tool [36], namely a VMD [37] plug-in, PROPKA2 [38], [39]. It is widely used for protein or protein/ligand pKa calculations and it is also embedded in various tools such as: CCG MOE (Molecular Operating Environment), PKD, PDB2PQR, WEBPDB and VEGA-ZZ. This fast and efficient software is based on empirical methods. With this method, good correlations were found with experimental data [38]–[40]. The method was found superior compared to other pKa calculation tools [40]. PROPKA2 calculates the pKa of a group through the combination of an environmental perturbation, ΔpKa, and the unperturbed pKa value of the group, pK_Model._

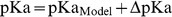
(1)where pK_Model_ and ΔpKa are determined empirically. For each ionizable group, ΔpKa consists of five terms including global (GlobalDes) and local desolvation (LocalDes), hydrogen bonds with side-chain groups (SDC-HB), hydrogen bonds with amide backbone (BKB- HB), and interactions with charged groups (ChgChg).
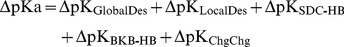
(2)


Simple distance functions and, for backbone hydrogen bonds, distance/angle functions with a constant empirical pKa shift are introduced to compute the above ΔpKa terms. This scheme neglects the effect of nonsigmoidal titration curves for final pKa values. However, this effect is generally smaller than 1.0 pH unit errors observed for most other pKa prediction methods [41]. All available GH32 and GH68 structures were used for these calculations.

### pKa calculations of the acid/base E203 in AtcwINV1 (complexes) and the transition state

**Figure 6:**
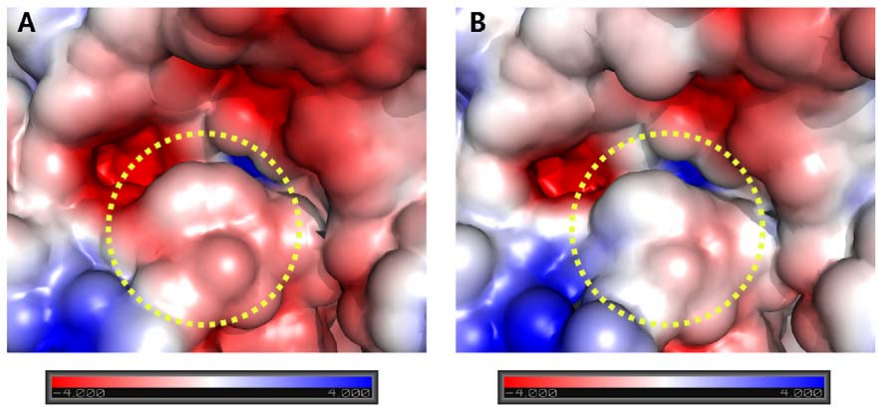
Electrostatic maps for AtcwINV1 and its D239A mutant. Electrostatic maps were generated by the APBS [55] tool in PYMOL. AtcwINV1 (Panel A, PDB: 2AC1) and its D239A mutant (Panel B, PDB: 2QQU) are presented. The region occupied by the D239A mutant (dotted circle) is much more hydrophobic than in the wild type.

pKa values for the acid-base catalyst E203 and its neighboring key residues R148 (**R**DP motif) and Y279 (**Y**ASK motif) were calculated for the AtcwINV1 apo-enzyme, the mutants in complex with sucrose, the apo-enzyme with docked sucrose and the transition state (Table 1). The protonation state ([A**^−^**]/[HA]) of the acid/base E203 is also indicated (Table 1), calculated at pH 5.0, the pH optimum of AtcwINV1 [23]. It can be concluded that E203 is only partially protonated in the apo-enzyme. Indeed, the ratio between deprotonated and protonated state [A^−^]/[HA] is 2.0. Sucrose entrance (docked sucrose in 2AC1) leads to a more complete protonation ([A^−^]/[HA] = 0.1), facilitating catalysis. A similar pKa increase of E203 was observed in the D23A mutant in complex with sucrose and for the transition state (Table 1). By contrast, the D239A mutant in complex with sucrose shows a dramatic decrease in the pKa value for E203 (i.e. [A^−^]/[HA] = 25.1), counteracting efficient catalysis. This result is in line with the poor catalytic properties of the D239 mutant [26]. D239 and K242 (or a homologue R residue) form a D/K or D/R couple (Figure 2) in GH32 enzymes using sucrose as a preferential donor substrate [12], [26]. So far it was assumed that mutating the amino acids, making up this couple, would drastically interfere with the sucrose binding as a donor substrate. The possibility to generate a D239A sucrose complex [30] suggests that at least partial sucrose binding is still possible. The results presented here suggest that the pKa value for E203 in D239A is so low that most of the E203 would be deprotonated (Table 1), leading to a very inefficient catalysis on (that part of) sucrose molecules that would still be able to bind. It should be noted that the D239A mutation greatly changed the active pocket electrostatic properties, making it more hydrophobic and less accessible for a water molecule acting as a final fructosyl acceptor (Figure 6). It can be concluded that the poor catalytic properties of the D239A mutant can be explained by three different reasons: (1) a reduced sucrose binding efficiency [26] (2) a decreased pKa of E203 and (3) a more hydrophobic active site affecting water entrance (Figure 6).

### pKa modulation and Molecular Dynamics on AtcwINV1

**Figure 7:**
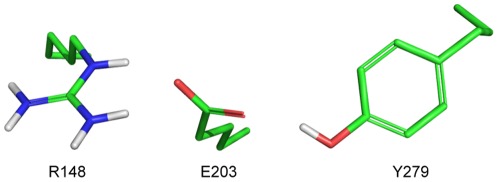
E203 interactions in AtcwINV1. E203 in AtcwINV1 (PDB: 2AC1) interacts with Y279 via its keto (C = O) function and with R148 through its C-O^−^ bond.

The pKa of R148 is generally 3 to 5 units lower than the one of Y279, which means that R148 is almost 10^3^–10^5^ times more acidic (Table 1) and as such able to release a proton more efficiently as compared to Y279. This strongly suggests that R148 functions as a pKa modulator, rather than the previously proposed Y279 residue (the homologue in Ci1-FEH IIa is Y274 [27]). Furthermore, 2AC1 structural refinement with Brugel [42] and visualization with PYMOL [43] showed that the C = O double bond in E203 is pointing to Y279 while the C-O**^−^** has the ability to accept a proton from R148 (Figure 7).

**Figure 8:**
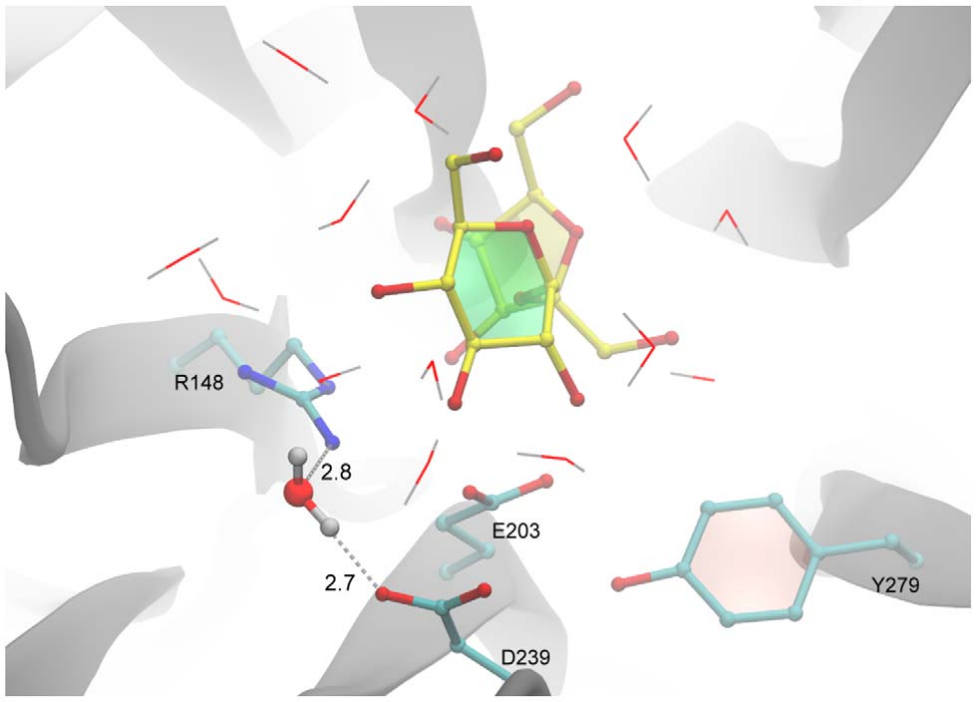
A water molecule between R148 and D239. A water molecule is frequently observed between R148 and D239 during MD simulations. Distance units are in angstrom (Å).

A 5ns MD simulation was pursued by program Desmond [44] for the docked AtcwINV1/sucrose complex. Intriguingly, a water molecule is frequently observed between R148 and D239 (Figure 8). At pH 5.0, the pH optimum of AtcwINV1, this water can be protonated (H_3_O^+^) and attracted to an area with negative electrostatic surface near the catalytic pocket (Figure 6). It can be speculated that this particular H_3_O^+^ could assist in stabilizing the position of D239. Additionally, this H_3_O^+^ could also act as a proton donor to R148, after R148 donated its own proton to E203, upon entrance of the substrate (see also below).

### pKa calculations on the acid/base E201 in Ci1-FEH IIa and its complex with sucrose as inhibitor. Comparison with the Aspargillus awamori exo-inulinase

In contrast to invertases, plant fructan exohydrolases (FEHs) use fructans as preferential donor substrates. For instance 1-kestose (a fructan trisaccharide Glc-Fru-Fru) and inulobiose (a fructan disaccharide Fru-Fru) are suitable substrates. Sucrose (Glc-Fru) is not a donor substrate. In many cases, FEHs are strongly inhibited by sucrose. This is also the case for 1-FEH IIa of *Cichorium intybus* (Ci1-FEH IIa) [23], the first plant GH32 enzyme of which the 3D structure was resolved [27]. The pH optimum of Ci1-FEH IIa is 5.0, similar to the one of AtcwINV1. The protonation state of E201 in the apo-enzyme (PDB: 1ST8) is low (Table 1), while entrance of the substrates 1-kestose and inulobiose results in a more complete protonation, allowing enzyme catalysis at pH 5.0. The docked complexes with 1-kestose and inulobiose are shown in Figure S2, indicating the involved H-bonds. Similar to the AtcwINV1- D239A mutant in complex with sucrose, the Ci1-FEH IIa-sucrose complex 2ADD shows a pKa value well below the pH optimum, preventing catalysis. Hitherto, the inhibitory effect of sucrose in 1-FEH IIa and the absence of catalytic activity on sucrose were explained by the rotated position of the Glc moiety, bringing O2 in a favorable position to make a hydrogen bond with E201, counteracting catalytic activity [27]. The results provided in this manuscript now offer an alternative point of view to explain the absence of catalysis when sucrose is provided to Ci1-FEH IIa.

**Figure 9:**
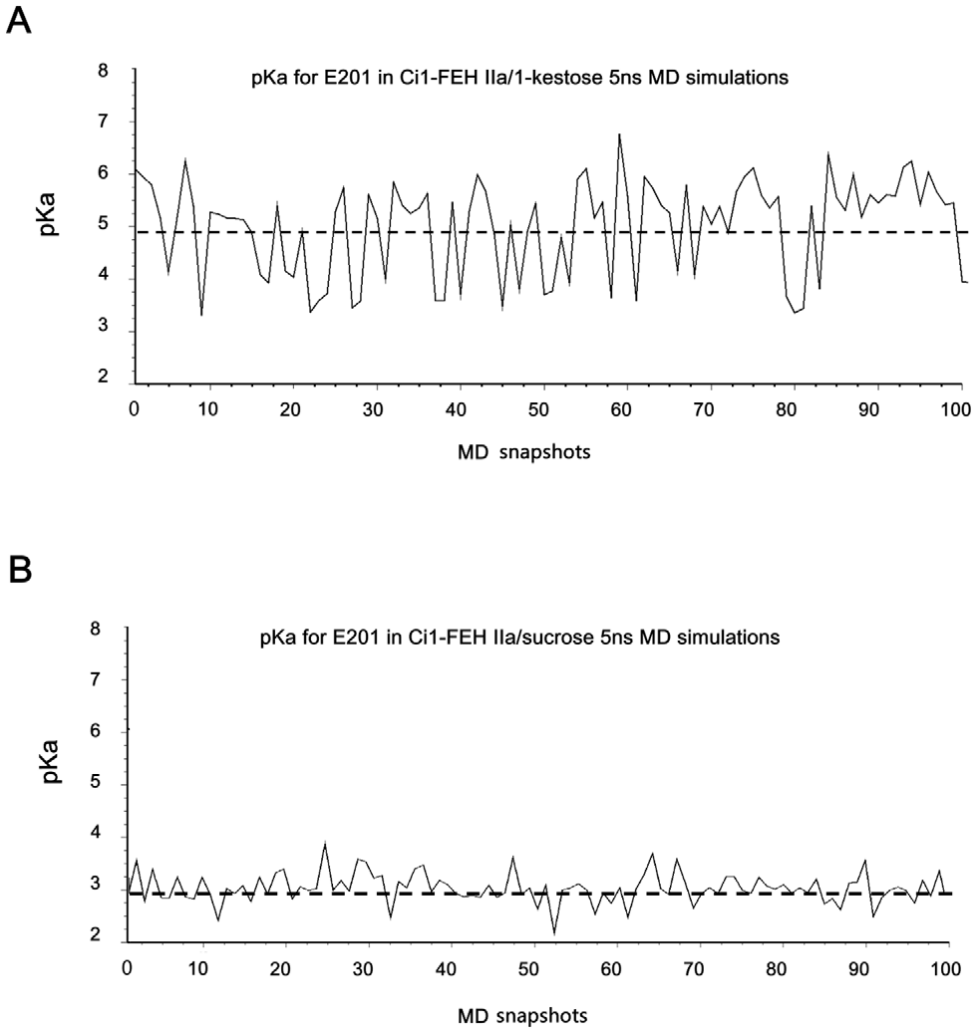
pKa fluctuation for 100 snapshots extracted from the trajectory throughout the 5 ns MD simulations. (A) pKa for E201 in 1-FEH IIa/1-kestose. Its mean (dash line) is 5.0 with standard deviation 0.88; (B) pKa for E201 in 1-FEH IIa/1-sucrose. The mean for this case is 2.9 with standard deviation 0.30.

To further corroborate this alternative hypothesis, pKa values were also evaluated on a conformational ensemble of two 5 ns MD simulates: 1-FEH IIa/sucrose and 1-FEH IIa/1-kestose. Over 100 snapshots throughout the whole simulation were extracted for pKa analysis (Figure 9). The mean for 1-FEH IIa/sucrose is 2.9 with standard deviation 0.30; while the mean for 1-FEH IIa/1-kestose is 5.0 with comparatively higher standard deviation 0.88 owing to the flexibility of 1-kestose and residues nearby (Figures S3C and S4C). Statistical analysis showed that PROPKA derived pKa values are stable throughout the MD simulations (Figure 9) and consistent with pKa values 3.3 for 1-FEH IIa/sucrose and 5.4 for 1-FEH IIa/1-kestose presented in Table 1. Moreover, RMSD calculations on both sucrose and 1-kestose complexes confirmed overall stability with minor fluctuations (Figures S3 and S4): the RMSF for E201 in both cases are below 0.4 Å. Additionally, plotting pKa values and experimental k_cat_ showed good correlation (Figure S5).

Both the plant enzymes AtcwINV1 and Ci1-FEH IIa are examples of GH-J members with well-defined preferential donor substrate specificity, towards sucrose and fructans, respectively. Clearly in these enzymes the evolutionary process is almost reaching the point of “unifunctionality” with only minor side activities present next to a clear, main activity. This is different in microbial GH-J members often showing less strict substrate specificities. For instance, it is well known that the exo-inulinase of *Aspergillus awamori* is not very specific to fructans, since it also accepts sucrose as a donor substrate, at least to some extent [17]. This substrate specificity fits nicely with the observed pKa values for the acid-base catalyst E241 of this enzyme and its complexes (Figure S2). Indeed, the apo-enzyme shows a pKa below the pH optimum, while the docking with sucrose shows a pKa close to the pH optimum and a docking with 1-kestose shows a pKa far above the optimal pH, promoting a more efficient catalysis (Table 1).

### Studies on other GH-J members

We extended our calculations to all available structures within the GH-J clan, listed in Table 1 (GH32) and Table 2 (GH68), respectively. First, it can be concluded that the acid/base is not fully protonated before the substrate enters the active site. The fructosyltransferase of *A. japonicus* (Table 1) is the only exception since its acid/base E292 is already protonated in the apo-enzyme. Maybe this is related to the presence of an unusual His residue (Figure S2) in its active site. Second, in all cases the pKa values of the acid/base increases upon substrate entry, this also holds true for the fructosyltransferase from *A. japonicus*. Third, the conserved Arg (RDP motif) next to the acid/base is usually much lower in pKa than the Tyr (YASK motif).

## Discussion

### Model for an extended catalytic mechanism within GH-J. A role for substrate binding and for a conserved Arg as pKa modulator?

**Figure 10:**
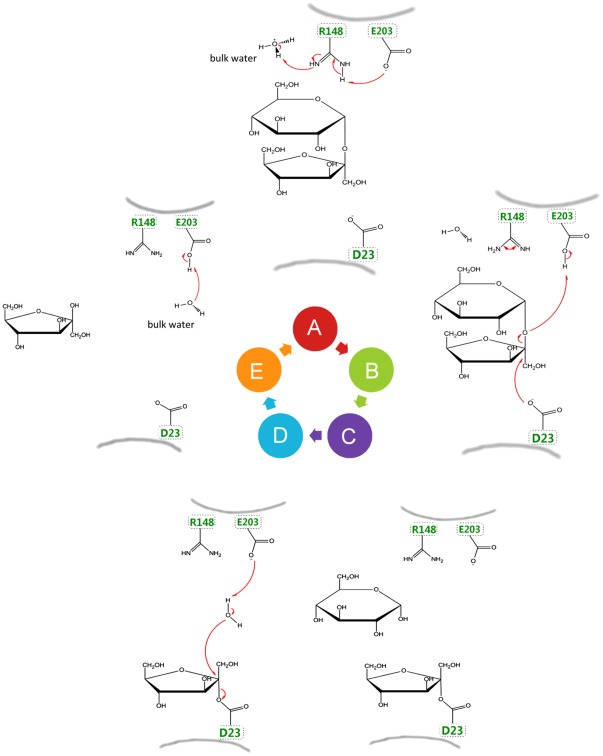
Proposed extensions on the reaction mechanism for the catalytic process in clan GH-J, as derived from AtcwINV1. (A) When sucrose binds in the active site of AtcwINV1, the pKa of E203 increases and it is protonated by a neighboring R148 which can extract a proton from a nearby water molecule (Figure 8) ; (B&C) the glycosidic oxygen in sucrose is protonated by acid/base E203, the nucleophile D23 attacks the anomeric carbon, forming a covalent enzyme-fructosyl complex and releasing glucose from the active pocket; (D&E) a water molecule is split, reprotonating the acid/base and leaving the hydroxyl group on fructose. The enzyme is ready to accept another sucrose molecule after fructose has left the active pocket.

The classic catalytic mechanism, based on original yeast invertase (GH32) research, assumes that the acid/base catalyst is sufficiently protonated to allow catalysis. Here, all (except one) pKa calculations predicted an insufficient protonation of the apo-enzymes. Therefore, we present here an extension on the original reaction mechanism (Figure 10). For simplicity, we focus on the AtcwINV1 and Ci1-FEH IIa cases, but comparable mechanisms might hold true for other GH-J members. In AtcwINV1, the acid/base E203 is only partially protonated before sucrose enters the active pocket. The pKa value of E203 increases when the substrate comes in and E203 will get protonated by R148, which on itself can extract a proton from a nearby H_3_O^+^ (Figure 10A). Next, the glycosidic oxygen in sucrose is protonated by acid/base E203, the nucleophile D23 attacks the anomeric carbon, forming a covalent enzyme-fructosyl complex and releases glucose from the active pocket (Figure 10B and Figure 10C). Finally, a water molecule is split, reprotonating the acid/base and leaving the hydroxyl group on fructose. During the leaving of the fructose moiety out of the active site the proton on E203 is mainly transferred to bulk water. (Figure 10D and Figure 10E). Upon the entrance of a new substrate the cycle will start again with the transfer of a bulk water proton to R148 and further to E203 (Figure 10A). In severe contrast, in case of Ci1-FEH IIa, the binding of sucrose does not increase the pKa, possibly explaining why sucrose acts as an inhibitor and not as a substrate for this enzyme.

These findings indicate that the pKa values of the active-site residues in an enzyme can be critical for the catalytic mechanism and actual substrate specificity. This is not merely limited within clan GH-J, but extends to many pH dependent enzymes including xylanases [45], amylases [46], lysozymes [47], proteinases [48], protein kinases [49] and others. Therefore, understanding pKa values of key role amino acids in the active site of such enzymes aims at understanding an array of biological processes and assists in the process of rational enzyme design. Various tools and methods focusing on pKa determinations have been developed for such purposes [50]–[52].

In summary, within the clan GH-J, most apo acid/bases are in a (partially) deprotonated state before substrate binding. The acid/base pKa value increases when a suitable substrate comes into the catalytic pocket. An acid/base neighboring and strictly conserved Arg, which can accept protons from the solvent environment, might transfer a proton to the acid/base. Thereafter, the protonated acid/base can further transfer the proton to the substrate for catalysis. When an inhibitor binds to the active pocket, the pKa value of acid/base is not increased, preventing catalysis. Favorite substrates seem to correlate well with a favorable increase of the acid/base pKa values. This strongly suggests that the exact binding modus of a sugar determines whether it will act as a substrate or as an inhibitor, by virtue of its effect on the pKa of the acid/base catalyst.

## Materials and Methods

### Protein Preparation

Structures were imported into the Maestro9.2 [53] program. All crystallographic solvent molecules and glycosyl chains were deleted. Hydrogens were added to the structure according to the pH environment as reported in literature. The protein preparation utility in Maestro9.2 was used to run a restrained minimization which removed unfavorable steric contacts and improved the quality of the protein hydrogen bonding network without large rearrangements of the protein heavy atoms.

### Docking

It is observed that the position of the fructose moiety is highly conserved within all the available crystal structures. Therefore, all oligosaccharides were initially placed such that the fructose moiety in the catalytic pocket is oriented in this conserved position. Cubic boxes centered on the ligand mass center with a size of 10 Å, 12 Å and 14 Å for the disaccharides, trisaccharides and tetrasaccharides, respectively, defined the docking binding regions. Extra-precision (XP) docking and scoring was executed in all cases. Twenty poses per ligand were included in post-docking minimization for bond length and angle as well as torsional angle optimization. Poses were rescored using a scaled Coulomb-van der Waals term and the GlideScore system. The best-scored poses were chosen as the optimal solution.

### Transition complex construction

The covalent transition state complex was built manually in Maestro9.2 [53] based on the previous docking result. The handmade transition state was submitted to Prime [54] in Maestro9.2 for energy minimization refinement, using the OPLS_2005 force field and Surface Generalized Born (SGB) continuum solvation model.

### pKa calculation

The prepared structures were submitted to PROPKA2 [38], [39] in VMD1.87 for pKa calculation. Default parameters were used throughout the calculations. Glycosyl chains were stripped off in all calculations to present the input data in the same way to the program. However, pKa values for structures with glycosyl chains were also performed but their values were not significantly different.

### Molecular Dynamics

All MD simulations were performed using the Desmond package, version 3.0.3. The OPLS_2005 force field was applied and topologies for the sucrose molecule were recognized automatically in Desmond. Each protein was placed in a trigonal box and solvated with TIP3P water molecules solvated with 0.15 M NaCl with a distance of 10 Å between any protein atom and the box edge, resulting in ≈55,000 atoms. Chloride counter-ions were added to compensate for the net positive charge of the protein. Unfavorable contacts in each system were relieved using 5000 steps of conjugate gradient energy minimization. A 100 ps length equilibration step at constant pressure (NPT ensemble) was executed with position restraints on all heavy protein atoms to allow relaxation of the solvent molecules. Next, a 5 ns production MD on each system was executed. During all simulations, pressure and temperature were coupled separately for protein and solvent atoms (including ions) to an external bath using the Berendsen coupling method at 298 K and 1 bar. The temperature and pressure constants were fixed to 0.1 ps and 1 ps, respectively. Periodic boundary conditions were imposed in all three directions. Short-range non-bonded interactions were cut off at 10 Å, while long-range electrostatic interactions were calculated using the particle-mesh Ewald (PME) summation schemes. The M-SHAKE algorithm was used to constrain all bonds in each system with a 2 fs integration step. Root Mean Square Deviation (RMSD) analyses were performed using the tools available in the Desmond suite.

## Supporting Information

Figure S1 **Conserved H-bond distances for AtcwINV1/sucrose MD simulations.** (A) Distance between D23(OD1) and sucrose(O1′); (B) Distance between D149(OD1) and sucrose(O4′); (C) Distance between W47(NE1) and sucrose(O6′); (D) Distance between D149(OD2) and sucrose(O3′). Distance units are in angstrom (Å).
(TIFF)

Figure S2 **Docked poses for clan GH-J members.** (A) Sucrose docked in exo-inulinase of *Aspergillus awamori*; (B) 1-kestose docked in exo-inulinase of *Aspergillus awamori*; (C) Crystal structure of sucrose in 1-FEH IIa of *Cichorium intybus*; (D) 1-kestose docked in 1-FEH IIa of *Cichorium intybus*; (E) Sucrose docked in β-fructosidase of *Thermotoga maritima;* (F) Sucrose docked in fructosyltransferase of *Aspergillus japonicus;* (G) 1-kestose docked in fructosyltransferase of *Aspergillus japonicus;* (H) Nystose docked in fructosyltransferase of *Aspergillus japonicus;* (I) Sucrose docked in fructofuranosidase of *Schwanniomyces occidentalis;* (J) Sucrose docked in β-fructofuranosidase of *Bifidobacterium longum;* (K) 1-kestose docked in β-fructofuranosidase of *Bifidobacterium longum;* (L) Sucrose docked in levansucrase of *Bacillus subtilis;* (M) Raffinose docked in levansucrase of *Bacillus subtilis;* (N) Sucrose docked in levansucrase of *Bacillus megaterium;* (O) Sucrose docked in levansucrase of *Gluconacetobacter diazotrophicus.*
(TIFF)

Figure S3 **RMSD (**Å**) and RMSF (**Å**) for 1-FEH IIa/sucrose MD simulations.** (A) RMSD of protein (1-FEH IIa) backbone; (B) RMSD of E201 heavy atoms with mean 0.24 and standard deviation 0.08; (C) RMSD of sucrose heavy atoms with mean 0.60 and standard deviation 0.18; (D) RMSF for each residue of 1-FEH IIa.
(TIFF)

Figure S4 **RMSD (**Å**) and RMSF (**Å**) for 1-FEH IIa/1-kestose MD simulations.** (A) RMSD of protein (1-FEH IIa) backbone; (B) RMSD of E201 heavy atoms with mean 0.36 and standard deviation 0.10; (C) RMSD of 1-kestose heavy atoms with mean 0.84 and standard deviation 0.21; (D) RMSF for each residue of 1-FEH IIa.
(TIFF)

Figure S5 **Correlation between pKa calculations and k_cat_.** X-axis, pKa calculation for AtcwINV1; Y-axis, **k_cat_** value for AtcwINV1 from experimental data according to Le Roy K, *et.al* 2007 [26]. The error bars for the pKa are estimations based on the calculated standard deviation average for the 2 MD runs shown in figure 9.
(TIFF)
