# Supplementary figures and images for: pKa Modulation of the Acid/Base Catalyst within GH32 and GH68: A Role in Substrate/Inhibitor Specificity?
Source: PLoS One. 2012 May 25;7(5):e37453. doi: 10.1371/journal.pone.0037453 (PMC3360783; doi:10.1371/journal.pone.0037453)

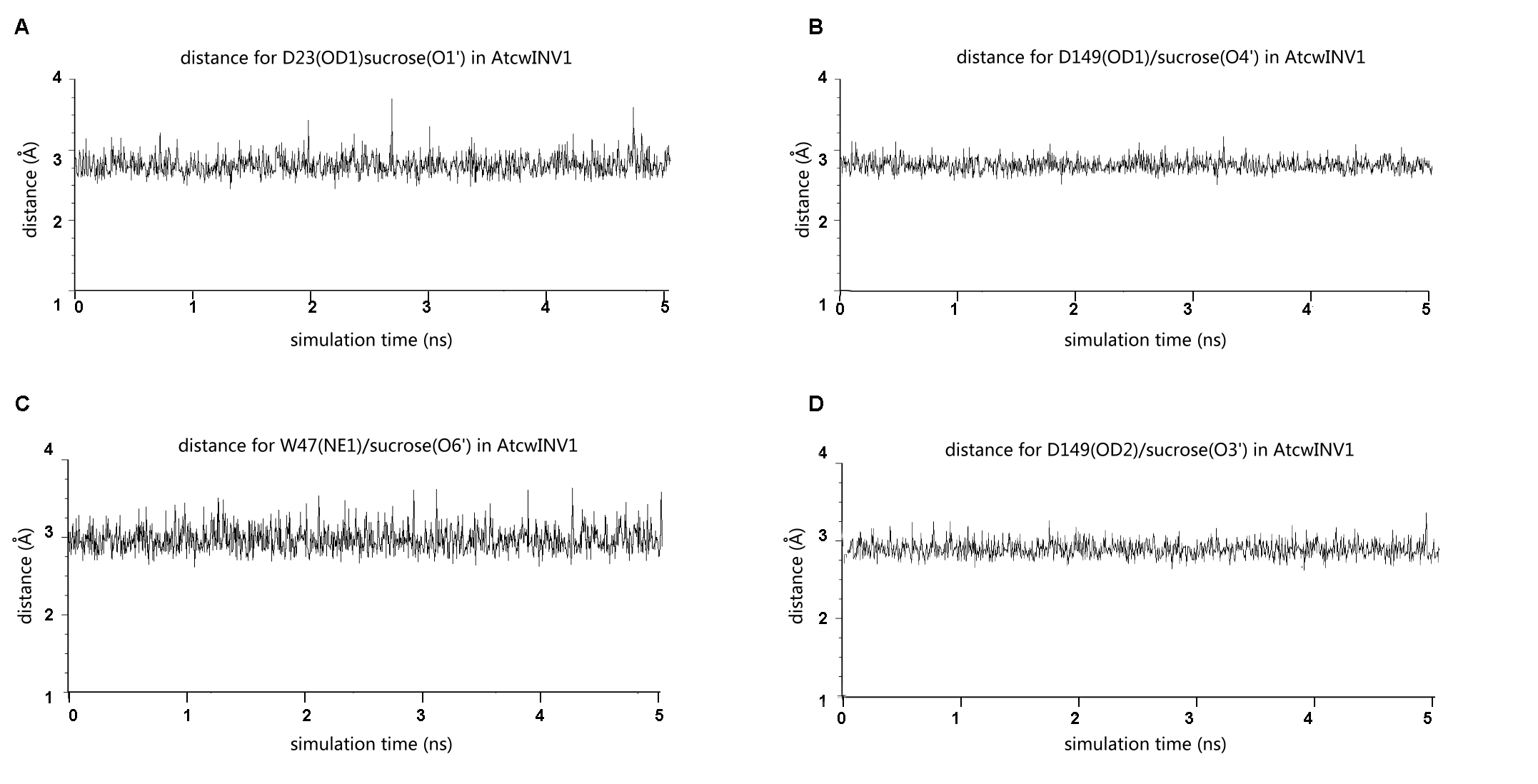

Supplement: Figure S1 — Conserved H-bond distances for AtcwINV1/sucrose MD simulations. (A) Distance between D23(OD1) and sucrose(O1′); (B) Distance between D149(OD1) and sucrose(O4′); (C) Distance between W47(NE1) and sucrose(O6′); (D) Distance between D149(OD2) and sucrose(O3′). Distance units are in angstrom (Å). (TIFF) [file pone.0037453.s001.tiff]

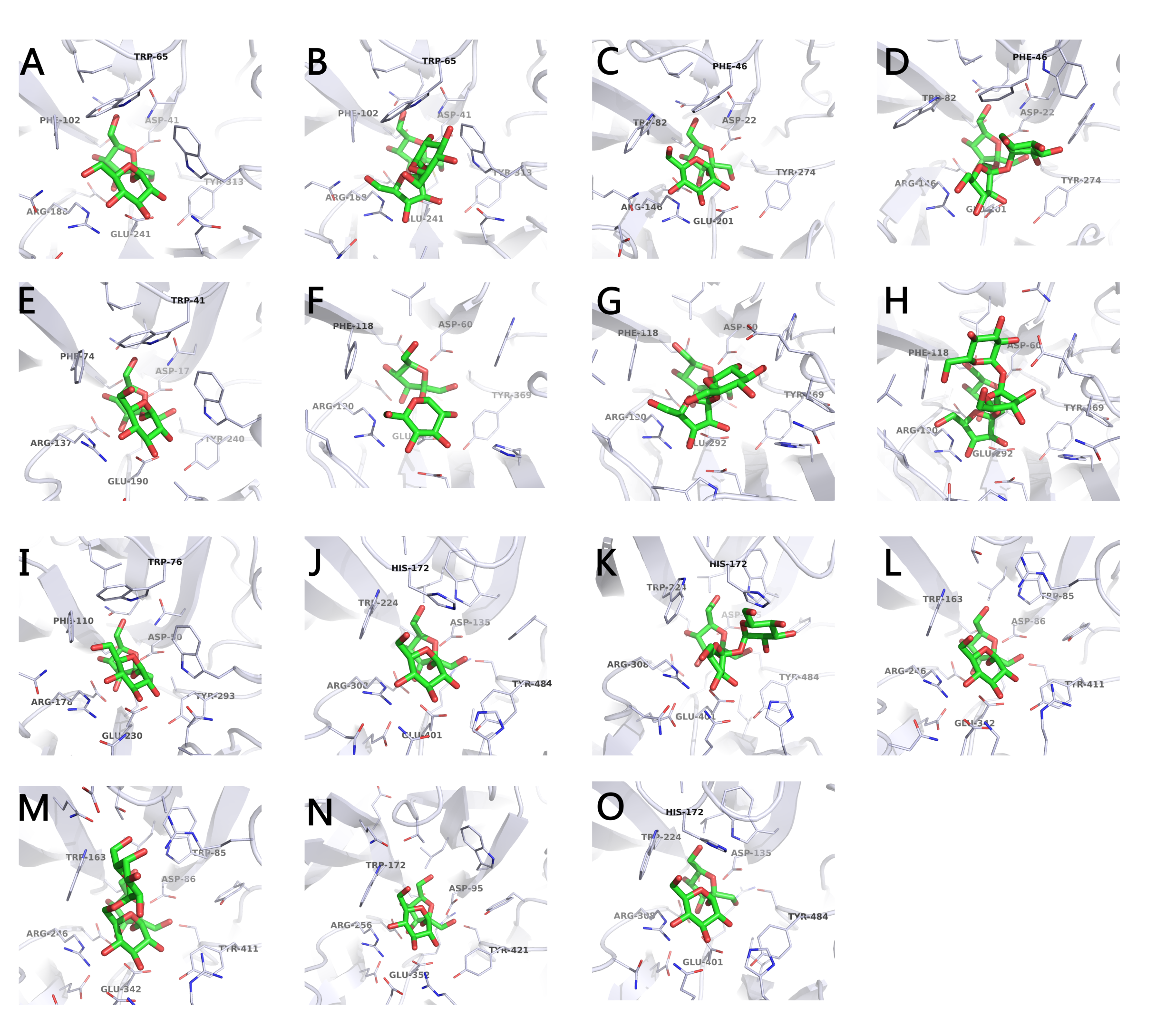

Supplement: Figure S2 — Docked poses for clan GH-J members. (A) Sucrose docked in exo-inulinase of Aspergillus awamori; (B) 1-kestose docked in exo-inulinase of Aspergillus awamori; (C) Crystal structure of sucrose in 1-FEH IIa of Cichorium intybus; (D) 1-kestose docked in 1-FEH IIa of Cichorium intybus; (E) Sucrose docked in β-fructosidase of Thermotoga maritima; (F) Sucrose docked in fructosyltransferase of Aspergillus japonicus; (G) 1-kestose docked in fructosyltransferase of Aspergillus japonicus; (H) Nystose docked in fructosyltransferase of Aspergillus japonicus; (I) Sucrose docked in fructofuranosidase of Schwanniomyces occidentalis; (J) Sucrose docked in β-fructofuranosidase of Bifidobacterium longum; (K) 1-kestose docked in β-fructofuranosidase of Bifidobacterium longum; (L) Sucrose docked in levansucrase of Bacillus subtilis; (M) Raffinose docked in levansucrase of Bacillus subtilis; (N) Sucrose docked in levansucrase of Bacillus megaterium; (O) Sucrose docked in levansucrase of Gluconacetobacter diazotrophicus. (TIFF) [file pone.0037453.s002.tiff]

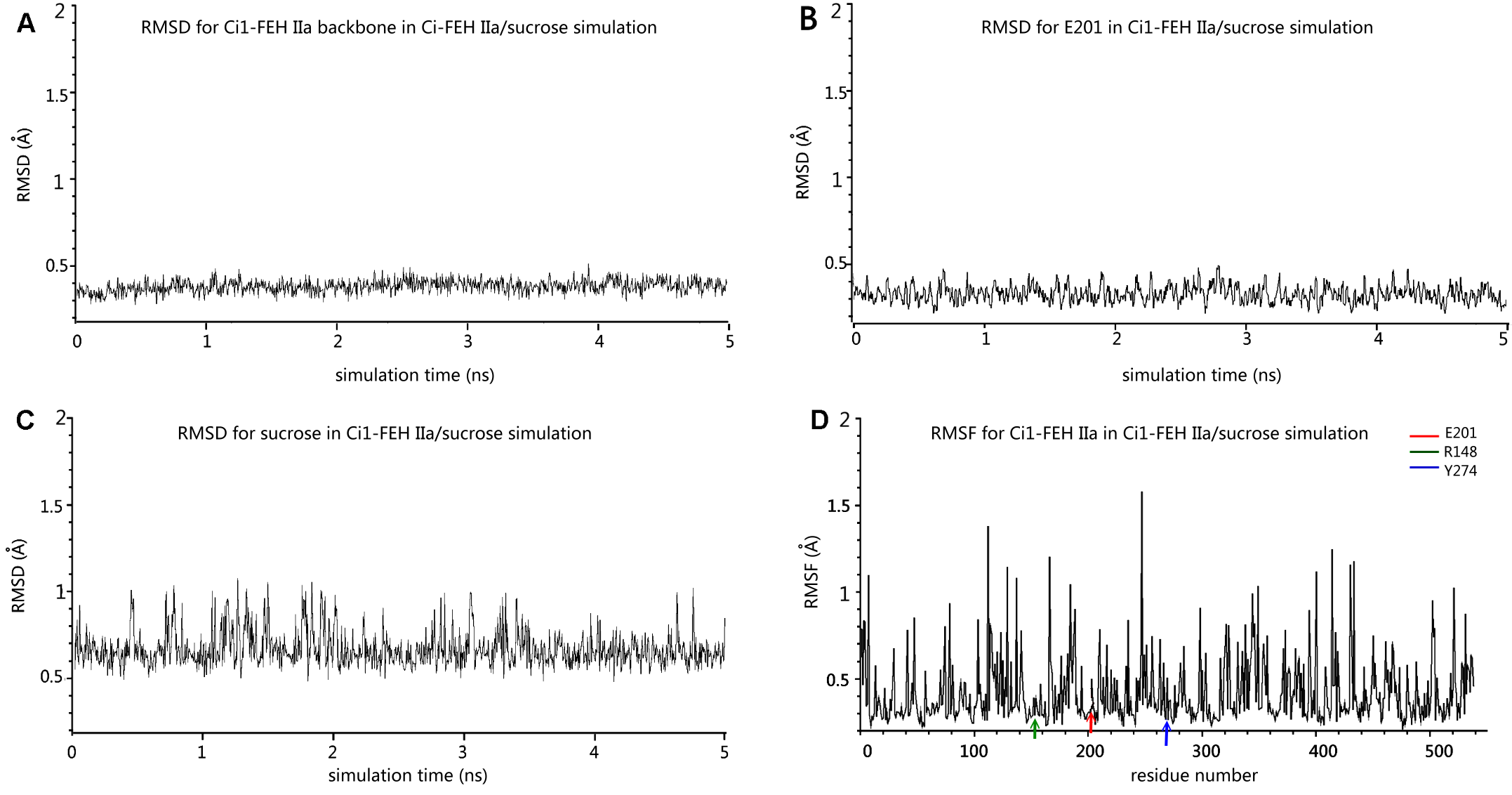

Supplement: Figure S3 — RMSD (Å) and RMSF (Å) for 1-FEH IIa/sucrose MD simulations. (A) RMSD of protein (1-FEH IIa) backbone; (B) RMSD of E201 heavy atoms with mean 0.24 and standard deviation 0.08; (C) RMSD of sucrose heavy atoms with mean 0.60 and standard deviation 0.18; (D) RMSF for each residue of 1-FEH IIa. (TIFF) [file pone.0037453.s003.tiff]

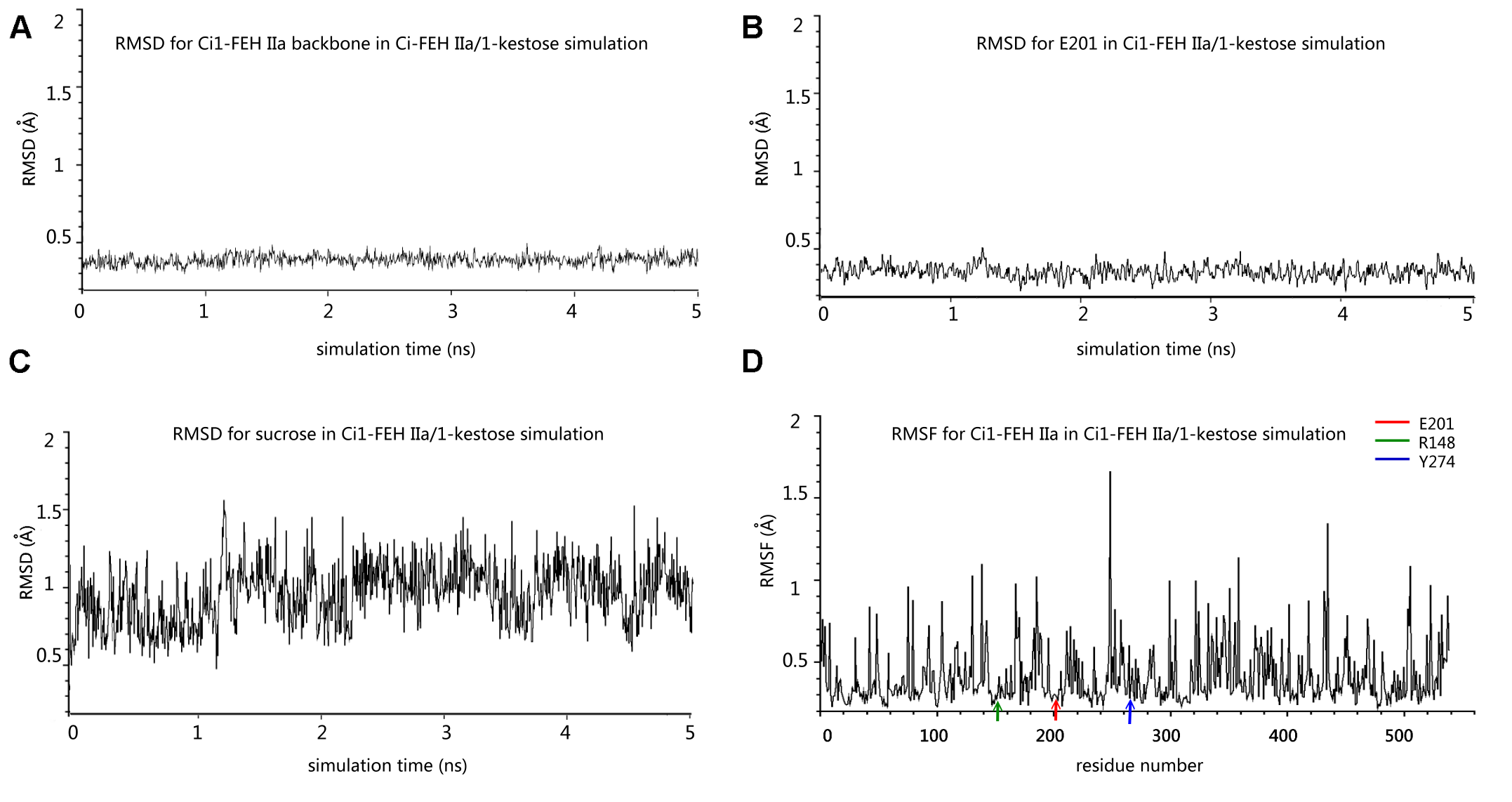

Supplement: Figure S4 — RMSD (Å) and RMSF (Å) for 1-FEH IIa/1-kestose MD simulations. (A) RMSD of protein (1-FEH IIa) backbone; (B) RMSD of E201 heavy atoms with mean 0.36 and standard deviation 0.10; (C) RMSD of 1-kestose heavy atoms with mean 0.84 and standard deviation 0.21; (D) RMSF for each residue of 1-FEH IIa. (TIFF) [file pone.0037453.s004.tiff]

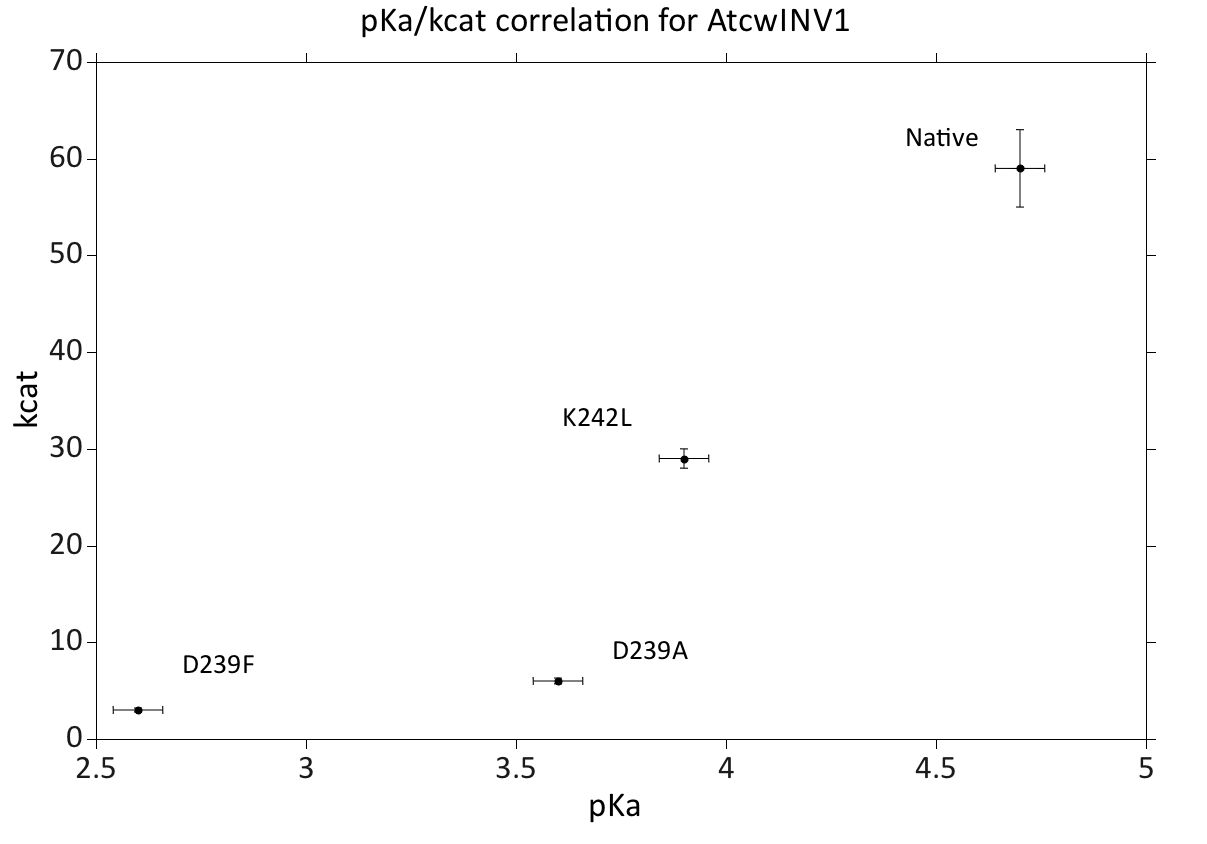

Supplement: Figure S5 — Correlation between pKa calculations and kcat. X-axis, pKa calculation for AtcwINV1; Y-axis, kcat value for AtcwINV1 from experimental data according to Le Roy K, et.al 2007 [26]. The error bars for the pKa are estimations based on the calculated standard deviation average for the 2 MD runs shown in figure 9. (TIFF) [file pone.0037453.s005.tiff]
